# Luxation Backward of the Internal Extremity of the Clavicle

**Published:** 1854-09

**Authors:** 


Luxation Backward of the Internal Extremity of the Clavicle.—*Reduction.*—John Combe, about 26 years of age, small and muscular, was brought to the hospital some moments after the following accident:—

He was walking in the street; he slips, falls forward, and strikes the anterior border of the clavicle against a stick of wood. Once on his feet, they were compelled to support him on each side to bring him to the hospital, two or three hundred yards distant.

The right shoulder was thrown backwards, and the neck was so bent forward that the chin rested on the sternum. The veins of the neck, and sternum temples were much dilated; the venous congestion rendered the mouth and lips livid. He staggered when he endeavored to walk, and responded with much difficulty to the questions proposed to him.

On raising the head, the nature of the accident was clearly seen.

The prominence of the sternal extremity of the clavicle was effaced. The direction of the bone could be traced with the fingers, from its acromial to its sternalextremity it inclined backwards to disappear behind the sternum.

I ordered an assistant to place his knee between the shoulders of the patient, to sever the anterior part of the shoulders and to maintain a vigorous traction backward. A second assistant steadied the head by placing his hands on the chin. At the same time, I endeavored to slip my fingers behind the clavicle, near the shoulder, to force the clavicle forward. This first effort was fully successful; a distinct sound advised us that the bone had resumed its normal position. The venous obstruction, and the semi-comatose state produced by the pressure of the clavicle upon the brachio-cephalic trunk disappeared, and in two or three minutes the patient had entirely recovered his senses and walked very well. A little swelling followed but it had almost disappeared, when the patient left our wards ten days afterwards.—*Edinburgh Med. and Surg. Jour.*

